# Fluoride in the Bones of Foxes (*Vulpes vulpes* Linneaus, 1758) and Raccoon Dogs (*Nyctereutes procyonoides* Gray, 1834) from North-Western Poland

**DOI:** 10.1007/s12011-014-0017-5

**Published:** 2014-05-30

**Authors:** Mirona Palczewska-Komsa, Elzbieta Kalisińska, Danuta I. Kosik-Bogacka, Natalia Lanocha, Halina Budis, Irena Baranowska-Bosiacka, Izabela Gutowska, Dariusz Chlubek

**Affiliations:** 1 Department of Biology and Medical Parasitology, Pomeranian Medical University, Powstanców Wlkp. 72, 70-111 Szczecin, Poland; 2 Department of Biochemistry and Medical Chemistry, Pomeranian Medical University, Powstanców Wlkp. 72, 70-111 Szczecin, Poland; 3 Department of Biochemistry and Human Nutrition, Pomeranian Medical University, Broniewskiego 24, 71-460 Szczecin, Poland

**Keywords:** Fluoride, Bone, Red fox, Raccoon dog, Bioaccumulation

## Abstract

Assessment of exposure to fluoride (F^−^) is increasingly focused on mineralized tissues, mainly bones. Their periodic growth and continuous reconstruction make them a good material for studying long-term F^−^ accumulation. In this study, F^−^concentrations were determined in the bones of foxes and raccoon dogs from north-western Poland and relationships between bone F^−^ and the age categories of the animals were attempted to be identified. Bone samples were collected from femurs of 32 foxes (15 males and 17 females) and 18 raccoon dogs (10 males and 8 females) from polluted, medium-polluted, and unpolluted by F^−^ areas. Bone F^−^ was determined by potentiometric method, and results were expressed per dry weight (dw); they ranged from 176 to 3,668 mg/kg dw in foxes and from 84 to 1,190 mg/kg dw in raccoon dogs. Foxes from north-western Poland accumulated much more F^−^ in their bones than raccoon dogs. Our study shows that the assessment of hazards created by industrial emitters can be conducted conveniently by the measurements of fluorine content in hard tissues of wild animals. Due to availability of such type of material for studies, it seems that the analysis of fluoride content in bones can be a good tool in the development of ecotoxicology.

## Introduction

Fluoride (F^−^), depending on the concentration, can have a moderately positive or toxic effect on living organisms. Among other things, F^−^ is involved in the biosynthesis of enzymes (adenylyl cyclase and HMG) and the mineralization of hard tissue and cartilage [1, 2]. Its deficiency may lead to disturbances in the binding of calcium, magnesium, and phosphorus in bones as well as hypomagnesaemia, both leading to bone demineralization. In contrast, excess F^−^ can cause fluorosis of the teeth, and in extreme cases, bone fluorosis and bone tumors [3–6]. An estimated toxic F^−^ dose for humans is 5 mg/kg body weight [7].

Ecotoxicological studies aiming at the indirect assessment of environmental pollution by various substances, for example F^−^, include the determination of concentrations in living organisms. Their concentrations are mainly determined in the organs responsible for the detoxification process, i.e., the liver and kidneys of mammals and birds. However, some of them, including F^−^, over time accumulate in increasing quantities in highly mineralized tissues. Therefore, for several decades, researchers have used this type of bone tissue to enable the assessment of long-term environmental pollution with F^−^ [8].

Biomonitoring of the environmental threat associated with F^−^ pollution is usually based on bone and tooth samples collected from herbivorous ungulates and small laboratory mammals. However, medium-sized omnivorous mammals seem to be more suitable, as their diet and longevity make is more similar to humans; these are, for example, Canidae: fox (*Vulpes vulpes*) and raccoon dogs (*Nyctereutes procyonoides*). Although both species have a number of traits that make them potentially good bioindicators, as mentioned by Apostoli [9], little data exists on the F^−^ concentrations in the mineralized tissues of foxes [10–12], and there is no data on the F^−^ concentration in the bones of raccoon dogs.

The fox (*Vulpes vulpes*, Linneaus, 1758) has wild populations in Eurasia, the northern part of Africa and North America, and areas of Australia and New Zealand [13, 14]. Foxes are omnivorous, but their diet is dominated by food of animal origin, such as voles, shrews, mice, moles, birds, chicks and eggs, occasional hares or rabbits, frogs, lizards, invertebrates, and carrion. In addition, the fox feeds on berries and fruits and is also often seen looking for food in garbage dumps and landfills [13, 15].

The raccoon dog (*Nyctereutes procyonoides*, Gray, 1834) comes from north-western Asia and inhabits areas of eastern Siberia, northern China, Manchuria, Korea, Japan, and the former Soviet Union [16]. Its diet includes small mammals (voles), carrion, fish, eggs, aquatic birds, molluscs, amphibians, reptiles, insects, earthworms, berries, grains, and plant shoots [15, 17–19]. This is an expansive species due to its high adaptability to environmental conditions and its omnivorous feeding habits. The raccoon dog was introduced into the Soviet Union in the 1920s, which led to a rapid migration to areas of western and northern Europe [16, 17, 20]; in Poland, the first raccoon dogs appeared in the 1950s [16, 21].

The aforementioned characteristics of the fox and raccoon dog (omnivorous diet, common occurrence, and presence in a given area throughout the year) predispose them to being potentially good bioindicators in the intermediate evaluation of environmental F^−^ pollution.

The aims of the study were to determine the F^−^ concentrations in femur compact bone of foxes and raccoon dogs from north-western Poland, to compare their bone F^−^ levels against background domestic and environmental conditions, and to identify possible existing relationships between the F^−^ concentrations in bones and the age categories of the analyzed animals. Moreover, a further aim of this study was evaluate the usefulness of the examined wild canids as bioindicators of environmental F^−^ pollution.

## Material and Methods

### Study Area

**Fig. 1:**
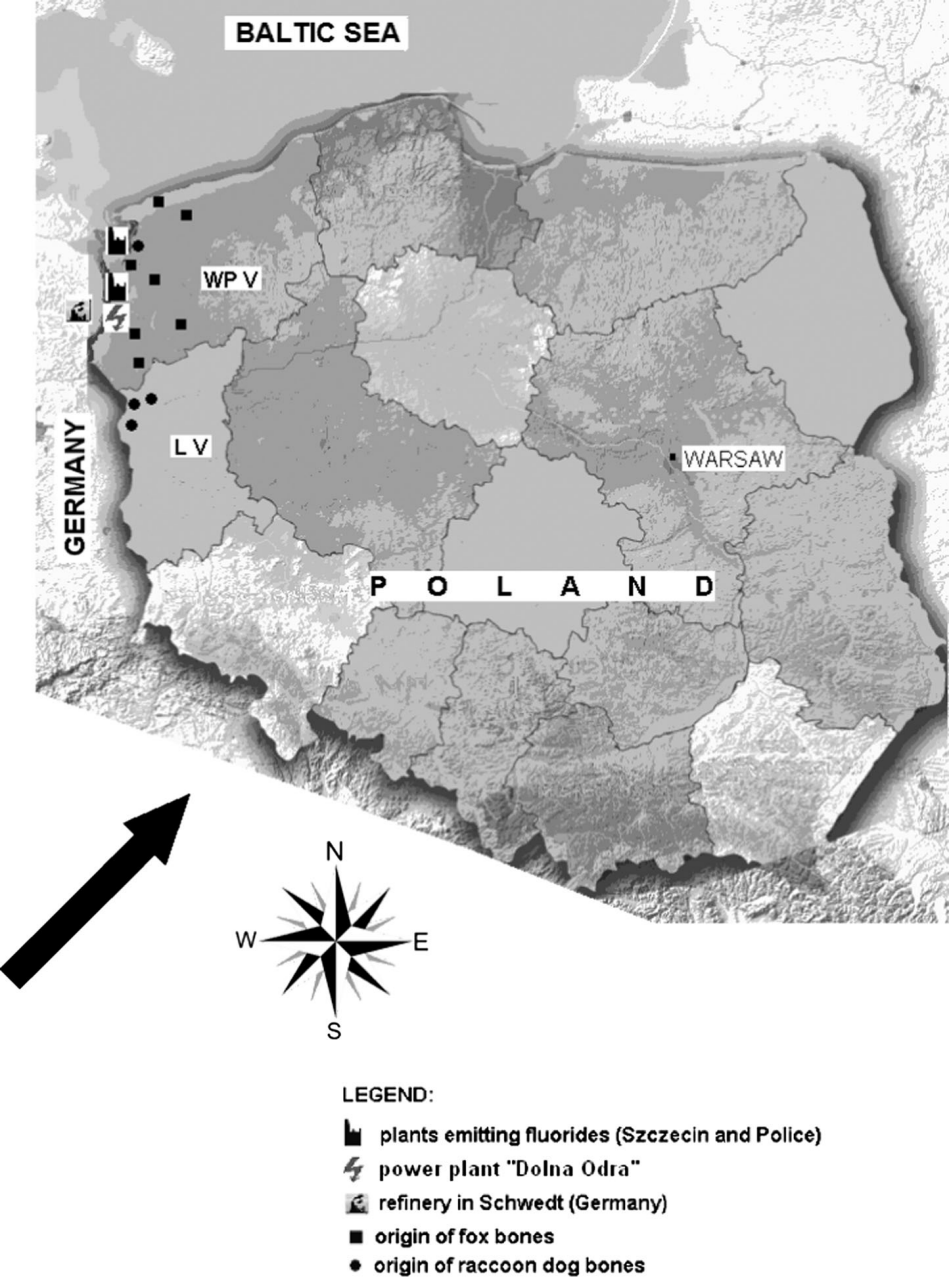
Map of Poland including the study area in West Pomeranian voivodship (*WP V*) and Lubuskie voivodship (*L V*) with plants, electricity, and refinery emitting fluorides and direction of predominant winds (*arrow*)

The material was collected in an area contaminated with F^−^ (<20 km from the source of F^−^ emission; polluted area); an area in the southern part of the West Pomerania, moderately polluted with F^−^ (>20 km from the source of F^−^ emission; medium-polluted area); and an area of the Warta Mouth National Park (WMNP, 8,074 ha), Lubuskie province (unpolluted area) (Fig. 1).

In the West Pomerania province, mainly its capital Szczecin (53° 25′ 57″ N, 14° 33′ 19″ E), there are or has been a number of industrial plants—sources of F^−^ emission into the environment (Fig. 1). In addition, there is also a refinery in Schwedt in Germany, located near the German-Polish border, which has no less impact on the environment than the plants in Poland, taking into account the prevailing winds in Western Pomerania. Furthermore, the F^−^ waste from the plants is often discharged to wastewater and into rivers and then into the drinking water. The Gunica River near the Police Chemical Plant SA has at least 10 times higher F^−^concentration compared to most Polish rivers [22, 23].

The area of Warta Mouth National Park (WMNP, 8,074 ha) is periodically inundated by the waters of the Warta and Odra Rivers (Polish/Czech: Odra; German: Oder). The Warta flows into the Odra near Kostrzyn (52° 35′ N; 14° 40′ S), a city in western Poland. The Odra is the second largest river in Poland, constituting a large section of the Polish-German border. River sediments collected from the Odra and Warta Rivers in the vicinity of Kostrzyn contain elevated levels of heavy metals, including Hg [24, 25]. The concentration of Hg in the sediments of the Odra ranged from 0.25 to 1.49 mg/kg in Kostrzyn [25].

### Material

Femurs were collected from 32 foxes (15 males and 17 females). The foxes were divided into two groups: foxes from a polluted (*n* = 10) and medium-polluted (*n* = 13) by F^−^ areas. The foxes were qualified into one of two age categories (immature or adult) according to measurements of canine teeth in accordance with the work of Knowlton and Whittemore [26]. The foxes were classified as immature (im) when aged 10–12 months (*n* = 16) and adult (ad) when over the age of 12 months (*n* = 16).

Femurs were also obtained from 18 raccoon dogs (10 males and 8 females). The animals were sourced from two groups: raccoon dogs from polluted (*n* = 4) and unpolluted (*n* = 14) by F^−^ areas. The raccoon dogs were divided into two groups: specimens weighing <4.5 kg (*n* = 8) were considered immature and with the weight >4.5 kg (*n* = 10) were classified as adult [27].

### Preparation of Material for Chemical Analysis and Determination of Fluoride

After the removal of remaining ligaments and muscles, the samples were stored frozen at −20 °C until analysis. In analysis, the compact bone was used. The samples were dried to constant weight in an oven at 105 °C. The percentage of water content in the samples was determined by gravimetric method. The dried samples were ground in an agate mortar. Samples weighing ~1 g were mixed with 1 ml of perchloric acid and shaken at 90 °C for 1 h. After cooling, 0.5 ml sample was transferred to a plastic tube, and then to 2 ml of sodium citrate solution and 2.5 ml of TISAB II. Reagent solutions were prepared with highly purified water (PURELAB Option Elga). Determination of the F^−^ concentration was performed by potentiometry with ion-selective Orion electrodes (Thermo Scientific, USA). F^−^ content in the sample was calculated based on the potential difference measured in each sample, the sample weight, and the concentration of the added standard. After mixing, the potential difference of each sample was measured for 10 min, 5 min before the addition of the appropriate standard and 5 min after the addition. Details of the analytical procedure are presented in the work by Gutowska et al. [28–30].

The correctness of the analytical procedure was controlled by determining the concentration of F^−^ in materials with known concentrations, i.e., standard NaF solutions at concentrations of 0.1, 1, and 10 mg/kg (Orion Company, USA).

### Statistical Analysis

Statistical analysis was performed using STATISTICA 10.0 software (StatSoft) and Microsoft Excel 2007. It included determination of the average F^−^ concentration in the bones of the examined species. Arithmetic means (AM), standard deviations (SD) from AM and medians (Med) were calculated. Compliance of distributions of F^−^ levels with normal distribution was checked using a Kolmogorov-Smirnov test with Lilliefors correction. The distribution of bone F^−^ concentrations in foxes and raccoon dogs was not normal (K-S = 0.2, *p* < 0.01 for foxes; K-S = 0.2, *p* < 0.05 for raccoon dogs). Therefore, further statistical analysis used a nonparametric Mann-Whitney *U* test (M-W U). All comparisons were only performed for groups where the number of specimens was *n* ≥ 4.

## Results

Comparison of bone F^−^ concentrations in samples collected from all animals showed a significant difference between the species (*U* = 161, *p* < 0.01). In foxes, it was approximately 64 % higher than in raccoon dogs (Table 1).

**Table 1:** The fluoride concentration (mg/kg dw) in the bones of the foxes and raccoon dogs by gender and age category

Gender, age category	Statistical parameters	F^−^ concentration in the bone		M–W U (foxes vs raccoon dogs)
		Foxes	Raccoon dogs	
M + F, imm + ad	AM ± SD	738.5 ± 600.1	446.6 ± 301.2	*U* = 161
	Med	607.9	371.6	
	Min–max	175.9–3668.1	83.7–1190.3	*p* < 0.01
	*n*	32	18	
M, imm + ad	AM ± SD	902.4 ± 802.4	340.3 ± 151.2	*U* = 15
	Med	752.8	363.9	
	Min–max	290.1–3668.1	103.2–598.0	*p* < 0.001
	*n*	15	10	
M, imm	AM ± SD	776.8 ± 234.0	330.7 ± 163.9	*U* = 8
	Med	772.0	363.9	
	Min–max	500.2–1182.7	103.2–492.0	*p* < 0.03
	*n*	6	4	
M, ad	AM ± SD	986.1 ± 1036.0	346.7 ± 157.9	*U* = 0
	Med	741.9	336.2	
	Min–max	290.1–3668.1	171.8–598.0	*p* < 0.01
	*n*	9	6	
F, imm + ad	AM ± SD	593.9 ± 295.2	579.6 ± 67.8	NS
	Med	542.7	480.2	
	Min–max	175.9–1105.9	83.7–1190.3	
	*n*	17	8	
F, imm	AM ± SD	643.0 ± 316.0	310.8 ± 215.3	NS
	Med	581.1	280.9	
	Min–max	175.9–1105.9	83.7–598.0	
	*n*	10	4	
F, ad	AM ± SD	523.9 ± 269.8	848.3 ± 348.7	NS
	Med	422.2	920.2	
	Min–max	329.6–1062.6	362.4–1190.3	
	*n*	7	4	
M + F, imm	AM ± SD	693.2 ± 269.8	320.8 ± 177.4	NS
	Med	681.2	333.8	
	Min–max	329.6–1062.6	83.7–598.0	
	*n*	16	8	
M + F, ad	AM ± SD	783.9 ± 810.8	547.3 ± 348.5	*U* = 16
	Med	536.5	411.7	
	Min–max	290.1–3668.1	171.8–1190.3	*p* < 0.004
	*n*	16	10	

*AM* mean, *SD* standard deviation, *Med* median, *n* number of individuals, *F* female, *M* male, *imm* immaturus, *ad* adultus, *M–W U*, Mann–Whitney *U* test, *p* significance level, *NS* nonsignificant

Comparisons were made within sex and age groups of the animals (Table 1). Statistically significant differences were found between male foxes and male raccoon dogs. The median bone F^−^ in samples taken from male foxes was about two times higher than in male raccoon dogs (*U* = 15, *p* < 0.001). Statistical differences were also found between young male foxes and young male raccoon dogs (*U* = 8, *p* < 0.03) and between adult individuals (including males and females) of foxes and raccoon dogs (*U* = 16, *p* < 0.004; Table 1).

F^−^ concentration range in the compact femoral bone of foxes was 175.9–3,668.1 mg/kg dry weight (dw). The greatest median F^−^ concentration was observed in young males and the lowest in adult females (Table 1), but a comparison using a M-W test showed no statistical significance.

The F^−^ concentration range in the femoral compact bone of raccoon dogs was in the range 83.7–1,190.3 mg/kg dw. The greatest median F^−^ was found in adult females and the lowest in young females (Table 1). In young females, raccoon dogs’ median F^−^ was about three times lower compared to adult females. Median F^−^ concentrations in the bones of adult males were 174 % lower compared to adult females

Comparing the two species of canids collected in the same area, a statistically significant difference in the bone F^−^ was recorded only in bones of foxes and raccoon dogs coming from polluted areas (*U* = 4, *p* < 0.03); median F^−^ concentration in the bones of fox proved to be more than two times lower in raccoon dog (Table 2).

**Table 2:** The fluoride concentration (mg/kg dw) in the bones of the foxes and raccoon dogs by origin

Origin	Statistical parameters	F^−^ concentration in the bone	
		Foxes	Raccoon dogs
Szczecin and surroundings (polluted area)	AM ± SD	978.3 ± 995.8	332.6 ± 92.0
	Med	747.4	336.2
	Min–max	290.1–3668.1	242.0–417.0
	*n*	10	4
Southern part of voivodship West Pomeranian (medium-polluted area)	AM ± SD	560.3 ± 268.3	–
	Med	500.2	
	Min–max	175.9–1001.4	
	*n*	13	
Voivodship Lubuskie (unpolluted area)	AM ± SD	–	479.2 ± 333.9
	Med		371.6
	Min–max		83.7–1190.3
	*n*		14

*AM* mean, *SD* standard deviation, *Med* median, *n* number of individuals

The bones of foxes coming from a polluted area were characterized by a greater F^−^concentration than foxes from medium-polluted area, and the difference was statistically significant (Table 2).

We also analyzed differences in bone F^−^ between raccoon dogs from polluted and unpolluted areas. Median F^−^ concentrations in the bones of raccoon dogs from both areas were similar, and no statistically significant difference was observed (Table 2).

## Discussion

The analysis of the content of chemical elements in tissues, like bones or teeth, is an important tool in toxicological and ecological examinations because of their ability to accumulate large quantities of elements including various trace elements and fluorine. Fluorine constitutes a part of fluoroapatites and hence stimulates both bone hardness and resilience [20, 21], as well as enamel solubility in teeth [18, 21].

In literature, F^−^ concentrations in bone are given per dry weight or in ash. To be able to make comparisons with our results (expressed in mg per kg dw), we used our own calculations and the works of Lanocha et al. [31] and Budis et al. [32] to convert units. It was assumed that the average water content in the compact bone of Canidae is 23 %. In most publications, bone F^−^ concentration is given as the arithmetic mean or a range of values, so in the comparison of our results with literature data, we use arithmetic means instead of medians.

There are few papers on bone F^−^ concentrations in wild canids. Kay et al. [33] analyzed F^−^ concentration in the bones of coyote *Canis latrans* in areas of North America not contaminated with F^−^. In coyotes from Montana (USA), the mean F^−^ concentration in the mandible was 321 mg/kg dw (Table 3), almost two times less than in the femurs of wild canids (foxes and raccoon dogs) from the polluted and medium-polluted areas in north-western Poland and 1.5 times lower than the bone F^−^ concentration in raccoon dogs from an unpolluted area (Warta Mouth National Park) observed in our study.

**Table 3:** The fluoride (F^−^) concentration (mg/kg) in wild canid bone material from various parts of the world

Place	Species	Tissue	Age	Gender	No. of animals	F^-^ (dw or ash)		Source
Poland, West Pomeranian voivodship	Fox (*Vulpes vulpes*)	Teeth, first permanent molars	>20 months, ad	F + M	14	dw	532 ± 169	[11]
						Ash	692 ± 224	
			<20 months, imm		20	dw	358 ± 208	
						Ash	461 ± 266	
Poland, Pomeranian voivodeship			<20 months, imm		7	dw	392	
						Ash	303	
Poland, West Pomeranian voivodship					13	dw	312	
						Ash	241	
Poland, West Pomeranian voivodship		Teeth, first permanent molars	6–20 months	F + M	4	dw	514 ± 309	[10]
						Ash	672 ± 401	
					10	dw	389 ± 242	
						Ash	510 ± 320	
Great Britain, Wales		Mandible	28.8 months	F + M	103	dw	551	[12]
Great Britain, Anglesey (excluding Holyhead)			16.6 months	F + M	52	dw	476	
Great Britain, Aberdeen TN			–	F + M	19	dw	283	
Great Britain, Anglesey, Holyhead TZ			–	F + M	8	dw	1,650	
USA, Montana	Coyote (*Canis latrans*)		–	–	2	dw	321	[31]

*F* female, *M* male, *ad* adult, *im* immaturus, *dw* dry weight

In Europe, research on bone F^−^ concentrations in canids was carried out in the UK by Walton [12], in mandibles of two groups of foxes living in areas with different degrees of F^−^ pollution. In foxes from uncontaminated areas, the mean F^−^ concentration in their mandibles was 283 mg/kg dw, several times smaller than in the mandibles of foxes inhabiting a nearby aluminum plant (Anglesey) which emits significant amounts of F^−^ (1,650 mg/kg dw) (Table 3). F^−^ concentration in the mandibles of foxes in the vicinity of Anglesy was almost 1.7 times higher than in the femurs of foxes (978 mg/kg dw) and almost 5 times greater than the mean F^−^ concentration in the femurs of raccoon dogs (333 mg/kg dw) from polluted area observed in our study. F^−^ level in the mandibles of foxes from areas not contaminated with F^−^ (Aberdeen) was 1.2 times smaller than in the bones of raccoon dogs obtained from unpolluted area in our study.

The differences we observed in F^−^ concentrations in the examined canids may have resulted from the following: interspecific differences F^−^ accumulation in tissues, a considerable differences in the number of animals, different habitats (including the degree of F^−^ contamination), diet, and type of bone material obtained for testing. The results showed north-western Poland is more contaminated with F^−^ than corresponding areas of the US and the UK, and the fox and raccoon dog exhibited a measurable response to the amount of F^−^ in the environment.

It should be noted that wild canids showed distinct differences in their bone F^−^. A statistically significant difference was found between foxes and raccoon dogs from polluted and medium-polluted areas; foxes showed more than 60 % greater F^−^ concentration. In addition, a statistically significant difference was noted between foxes and raccoon dogs collected in the same area (polluted area); the mean F^−^ concentration in the bones of foxes was almost three times higher compared to raccoon dogs (978 and 333 mg/kg dw, respectively). These results can be attributed to a different lifestyle and diet of these animals. The raccoon dog, as the sole representative of the Canidae, experiences anabiosis in response to an insufficient quantity of food in the winter. In contrast, the fox intakes food throughout the year, constantly accumulating F^−^ also in winter.

Bone F^−^ in the foxes living in north-western Poland has also been researched by Kalisińska and Palczewska [10] and Kalisińska and Palczewska-Komsa [11]; the samples were collected from the first molar mandibular teeth in foxes. Kalisińska and Palczewska compared F^−^ concentrations in two groups of foxes—hunted west of Szczecin (group I) and north of Szczecin (group II); in the former group, F^−^ concentration in the teeth (514 mg/kg dw) was more than 30 % higher than in the latter (389 mg/kg dw) [10]. Kalisińska and Palczewska-Komsa [11] compared, among others, a group of foxes from the Pomeranian province and a slightly more polluted West Pomeranian province. They observed low differences in F^−^ in the teeth of foxes from Pomeranian and West-Pomeranian voivodships (303 and 241 mg/kg dw, respectively), although they were not statistically significant. Foxes living in the north-western Poland (all individuals aged <20 months in both provinces) had similar mean F^−^ concentration in their teeth (almost 300 mg/kg dw), which may indicate a more or less uniform level of F^−^ contamination in this part of Poland (Table 3). That value is similar to F^−^ concentration found in the femurs of young raccoon dogs in our study (321 mg/kg dw), but more than two times lower than F^−^ in the femurs of young foxes (693 mg/kg dw). Both the teeth and bones of canids were collected in approximately the same period, and the differences are probably mainly due to differences in the chemical structure and composition of the tissues.

Indirect assessment of environmental F^−^ pollution is usually based on the analysis of bone samples (especially the mandible) of long-lived large ungulate mammals, which are generally herbivorous [34–37]. In those animals, bone F^−^ depends on the area of occurrence and their age, and to a lesser extent, on the species and the type of bone selected for analysis. As Western Pomerania has chemical plants using minerals containing significant amounts of F^−^ for the production of phosphoric acid and phosphate fertilizers (mainly Police and Szczecin), these areas are suitable for biomonitoring research of F^−^, usually carried out on cervids [34–37]. Gutowska et al. [34, 35] found that deer (*Cervus elaphus*) and roe deer (*Capreolus capreolus*) from areas remote from Szczecin agglomeration (Szczecinek, Połczyn Zdroj, and Swidwin) had bone F^−^ concentrations not exceeding 300 mg/kg dw.

Zakrzewska et al. [36] conducted a study on the bones of deer from north-western Poland in the late 1990s, i.e., at the beginning of modernization of chemical plants in Police. Bone samples were collected from animals in an area more exposed to F^−^ (I, western part of West Pomerania, including the areas of Szczecin and Police districts) and areas with much lower industrial pollution (II, the eastern part of the province). In deers originating from area II, bone F^−^ levels were usually 20 % lower (~245 mg/kg dw) compared to specimens in area I (~435 mg/kg dw). In our study, the mean bone F^−^ concentrations in the bones of wild canids from the north-western Poland (738 mg/kg dw in fox and 447 mg/kg dw in the raccoon dog) were higher than in deers investigated by Zakrzewska et al. [36] at least two decades previously. The results of these interspecific comparisons are in line with the observations of Kay et al. [33], who suggested that the omnivorous mammals (understood as an ecological group) accumulate more F^−^ than herbivorous mammals.

We also analyzed the relationship between bone F^−^ levels and the age of canines. Both Walton [12], who showed a correlation between bone F^−^ and the age of foxes (*r* = 0.59, *p* < 0.001) and Kalisińska and Palczewska [10] and Kalisińska and Palczewska-Komsa [11], who documented a relationship between fox tooth F^−^ and age (*r* = 0.38, *p* < 0.03), confirm this general regularity in long-lived mammals. However, in this study, although young foxes and raccoon dogs did have lower bone F^−^ (693 and 321 mg/kg dw, respectively) compared to adults of both species (738 and 547 mg/kg dw, respectively), the difference was statistically significant. Ultimately, the correlation between bone F^−^ concentration and the age of foxes and raccoon dogs turned out to be insignificant. One reason for this result could be a very large span of F^−^ concentrations in the bones of the examined specimens.

Summarized, uncontrolled uptake of fluoride released by industry represents a significant health problem both for humans and animals living in polluted areas [38]. Our study shows that the assessment of hazards created by industrial emitters can be conducted conveniently by the measurements of fluorine content in hard tissues of wild animals. Due to the availability of such type of material for studies, it seems that the analysis of fluoride content in bones can be a good tool in the development of ecotoxicology.
